# Low Prevalence of Clinically Significant Endoscopic Findings in Outpatients with Dyspepsia

**DOI:** 10.1155/2017/3543681

**Published:** 2017-01-22

**Authors:** Khaled Abdeljawad, Antonios Wehbeh, Emad Qayed

**Affiliations:** ^1^Department of Medicine, Division of Digestive Diseases, Emory University School of Medicine, Atlanta, GA, USA; ^2^Department of Medicine, Emory University School of Medicine, Atlanta, GA, USA

## Abstract

*Background.* The value of endoscopy in dyspeptic patients is questionable.* Aims.* To examine the prevalence of significant endoscopic findings (SEFs) and the utility of alarm features and age in predicting SEFs in outpatients with dyspepsia.* Methods.* A retrospective analysis of outpatient adults who had endoscopy for dyspepsia. Demographic variables, alarm features, and endoscopic findings were recorded. We defined SEFs as peptic ulcer disease, erosive esophagitis, malignancy, stricture, or findings requiring specific therapy.* Results.* Of 650 patients included in the analysis, 51% had a normal endoscopy. The most common endoscopic abnormality was nonerosive gastritis (29.7%) followed by nonerosive duodenitis (7.2%) and LA-class A esophagitis (5.4%). Only 10.2% had a SEF. Five patients (0.8%) had malignancy. SEFs were more likely present in patients with alarm features (12.6% versus 5.4%, *p* = 0.004). Age ≥ 55 and presence of any alarm feature were associated with SEFs (aOR 1.8 and 2.3, resp.).* Conclusion.* Dyspeptic patients have low prevalence of SEF. The presence of any alarm feature and age ≥ 55 are associated with higher risk of SEF. Endoscopy in young patients with no alarm features has a low yield; these patients can be considered for nonendoscopic approach for diagnosis and management.

## 1. Introduction

Dyspepsia is defined as chronic or recurrent pain or discomfort centered in the upper abdomen [1]. It involves a variety of symptoms such as epigastric pain or burning, early satiety, bloating, upper abdominal fullness, or nausea [2, 3]. Functional dyspepsia is defined by the Rome IV consensus as the presence of one or more of the following: bothersome postprandial fullness, bothersome early satiation, or bothersome epigastric pain or burning, with no evidence of structural disease to explain the symptoms. The criteria should be fulfilled for the last three months with symptom onset at least six months before diagnosis [4]. Dyspepsia is one of the most commonly encountered gastrointestinal complaints in the outpatient and inpatient settings. It is estimated that around 25–35% of the US population are affected by dyspepsia [3, 5, 6]. Dyspepsia has huge economic costs to patients and to the healthcare system. Patients with dyspepsia have lower work productivity and more sick leaves [7–10]. The approach for evaluating and managing patients with dyspepsia focuses on identifying high risk patients including those older than 55 years and those with one or more alarm features (bleeding, anemia, early satiety, unexplained weight loss, dysphagia, odynophagia, vomiting, family history of gastrointestinal cancer, previous esophagogastric malignancy, previous documented peptic ulcer, previous upper gastrointestinal surgery, lymphadenopathy, or an abdominal mass). It is recommended that these two groups of patients undergo Esophagogastroduodenoscopy (EGD) to exclude an organic pathology such as esophagogastric malignancy and peptic ulcer disease. Otherwise, patients can be managed by either the “test and treat” strategy for* H. pylori* or a trial of proton pump inhibitor (PPI) depending on the* H. pylori* prevalence [1, 11]. The yield of endoscopy in patients with dyspepsia is questionable and varies among studies; part of this variation is due to different definitions of dyspepsia used by different studies [6, 12]. A systematic review by Ford et al. examined studies that reported prevalence of endoscopic findings in outpatients with dyspepsia. A clinically significant finding was defined as erosive esophagitis, Barrett's esophagus, gastric or duodenal ulcer disease, and gastroesophageal malignancy. The pooled prevalence of significant endoscopic findings was 27.5% when using a broad definition of dyspepsia or 18% when including studies using the Rome criteria to define dyspepsia [13]. The most common clinically significant finding encountered was erosive esophagitis (20%) when using broad definition of dyspepsia or peptic ulcer disease (11%) when using the Rome criteria to define dyspepsia. Most of the included studies used a questionnaire to screen for eligible patients instead of medical staff evaluation, and in some studies all patients underwent endoscopy regardless of symptoms, age, or presence of alarm features [12, 14–18]. One study only included patients with positive* H. pylori* infection [19]. Those factors could have led to a higher prevalence of significant endoscopic findings. The utility of alarm features and age cutoff of 55 years in predicting the presence of significant endoscopic findings is unknown [20–22]. Furthermore, a large proportion of low risk patients with dyspepsia defined as younger than 55 and with no alarm features do not receive a trial of PPI or* H. pylori* testing prior to endoscopy [23]. In addition, it seems that many primary care physicians and even gastroenterologists do not define dyspepsia correctly and do not adhere to dyspepsia guidelines [24]. Due to the high prevalence of dyspepsia, a prompt endoscopy for every dyspeptic patient is not a practical approach, as this will lead to high costs and low yield of endoscopy [12, 25–27].

This study provides further clarification on the prevalence of significant endoscopic findings in outpatients with dyspepsia. It also evaluates the role of age and alarm features in clinical decision making regarding which patients should be referred to endoscopy. The primary aim of this study is to investigate whether age ≥ 55 and/or presence of any alarm feature predicts the presence of significant endoscopic findings (SEFs) in outpatients with dyspepsia at a large teaching hospital.

## 2. Methods

This is a retrospective study using the endoscopic procedure database at Grady Memorial Hospital in Atlanta, Georgia. This database prospectively collects information about all endoscopic procedures performed at the Gastroenterology unit, including procedure type, patient's medical record number, age, race, sex, procedure, indications, and findings. The study was approved by the Institutional Review Board. Inclusion criteria included all upper endoscopies performed in the outpatient setting for patients who were at least 18 year old and referred for dyspepsia between June 1, 2011, and July 1, 2015. Endoscopy referrals for patients with upper GI symptoms are made through the GI clinic, emergency department, primary care, and subspecialty physician offices. In our practice, most patients are seen in the GI clinic before their endoscopy. There are no specific referral criteria to the GI clinic. The decision to refer patients from the GI clinic to undergo upper endoscopy is at the discretion of the clinic physicians, based on age, severity of symptoms, and response to prior treatment. The medical record was carefully reviewed and the presence of dyspepsia symptoms (nausea, vomiting, epigastric pain/discomfort, postprandial fullness, belching, and early satiation) was recorded. Patients with heartburn and/or regurgitation were included only if they had accompanying dyspeptic symptoms. The medical record was also used to confirm endoscopic findings and collect further information about patients such as alcohol consumption, smoking status, and pertinent medications such as NSAIDs, PPI, H2-blockers, anticoagulants, ASA, and other antiplatelets.* H. pylori* infection status prior to endoscopy was recorded. At our institution, this is tested with the stool antigen test or serum antibody. Alarm features were recorded: (vomiting, weight loss, dysphagia, odynophagia, bleeding, anemia, early satiety, personal or family history of upper GI cancers, history of peptic ulcer disease, lymph node enlargement, or abdominal mass). Endoscopic findings were recorded in detail. We defined significant endoscopic findings as the presence of any of the following findings: gastric ulcer, duodenal ulcer, erosive esophagitis (LA grade B and higher), malignancy, stricture, or other findings that required specific therapy and were judged to have contributed to the patient's symptoms.

### 2.1. Statistical Analysis

Descriptive statistics were used to characterize patient demographic features. Continuous variables were summarized using mean and standard deviation, and categorical variables were summarized using number and percentage. We also categorized age as ≥55 and <55 years. We compared the presence of endoscopic findings in patients with and without alarm features and in patients within different age categories. The Chi-square test of independence was performed to examine the association of different endoscopic findings with the presence of alarm features. To examine the combined effect of age and presence of any alarm features on the presence of significant endoscopic findings, multivariate logistic regression was performed to examine the association of different factors (presence of any alarm feature, age ≥ 55, smoking, race, gender, PPI use,* H. pylori* status, NSAIDs, and alcohol) with the presence of significant endoscopic findings. Backward elimination was performed to remove nonsignificant covariates with a *p* value of >0.05.

## 3. Results

During the study period, 16,020 endoscopic procedures were performed, of which there were 4501 EGDs. Of those, 650 were performed for outpatients with dyspeptic symptoms and were included in the analysis.  Table 1 shows the basic demographics of the study population. The average age was 48.4 years ± 12.6. Two-thirds of the patients were younger than 55 years; 473 (72.8%) were females; 423 (65.1%) were African Americans; 161 (24.8%) patients were smokers and 65 (9.5%) used alcohol heavily. Among all patients, 504 (77.5%) were using Nonsteroidal Anti-Inflammatory Drugs (NSAIDs) at the time of the procedure or preendoscopic clinic visit, and 456 (70.2%) patients were on a PPI. Aspirin was used by 114 (17.5%) patients.* H. pylori* status was unknown in 350 (53.8%) of the patients, positive and treated prior to endoscopy in 140 (21.5%) patients, negative in 126 (19.4%) patients, and positive and untreated prior to endoscopy in 34 (5.2%) of patients.

The most encountered dyspepsia symptom was epigastric pain (76.6%), followed by nausea (43.1%) and vomiting (26.2%). Among all patients, 65.7% had one or more alarm features. Vomiting was the most common alarm feature (26.2%), followed by weight loss (21.2%) and anemia (15.8%). Of note, 28 (4.3%) patients had prior upper GI surgery, such as Billroth I and II and Nissen fundoplication. There were no reported major complications or deaths related to endoscopy during the study period.

### 3.1. Endoscopic Findings

Table 2 shows the findings of endoscopy stratified by the presence or absence of alarm features. Among all patients, 321 (49.4%) had any endoscopic abnormality. This did not statistically differ between patients with alarm features versus no alarm features (48.7% versus 50.7%, resp., *p* = 0.63). Only 66 (10.2%) patients had significant endoscopic findings. This was more likely to be found in patients with alarm features compared to those without any alarm features (12.6% versus 5.4%, *p* = 0.004). The most common endoscopic abnormality was nonerosive gastritis (29.7%), followed by nonerosive duodenitis (7.2%) and Los Angeles class A esophagitis (5.4%). Peptic ulcer disease was found in 26 (4%) of patients. This was more likely to be found in patients with alarm features compared to those without any alarm features (5.4% versus 1.3%, *p* = 0.01). Malignancy was found in only 5 (0.8%) patients, all of whom had one or more alarm features. Two patients had gastric adenocarcinoma, one had GIST tumor, one had MALT lymphoma, and one had squamous cell carcinoma of the esophagus.

Other SEFs were found in 25 (3.8%) patients. There were no significant differences in the presence of other SEFs between patients with and without alarm features (4.7% versus 2.2%, resp., *p* = 0.12). Other nonsignificant endoscopic findings (benign polyps and nonobstructive Schatzki's ring) were found in 6% of patients, and they were similar in distribution between patients with and without alarm features (6.7% versus 5.2%, resp., *p* = 0.41).

### 3.2. Significant Endoscopic Findings according to Age

SEFs in patients with and without alarm features as stratified by age are shown in Table 3. Older patients had a higher likelihood of having significant endoscopic findings. The prevalence of endoscopic abnormalities in patients without alarm features younger than 55 years was low (7/156, 4.5%), with the lowest prevalence in those younger than 40 years (1/64, 1.6%). The presence or absence of alarm features was predictive of SEFs among the main age categories (<55, ≥55).

Multivariable logistic regression analysis showed that age ≥55, presence of any alarm feature, and smoking were significantly associated with the presence of SEFs (Table 4). Having more than one of these risk factors significantly increases the chance of SEFs. Race, gender, PPI use prior to endoscopy,* H. pylori* status, NSAIDs, and alcohol use were not associated with SEFs.

## 4. Discussion

In this study, we found a low prevalence of significant endoscopic findings (10.2%) in outpatients with dyspepsia, and the majority of these were found in patients with alarm features. The prevalence of malignancy was extremely low (5 cases, 0.8%), and all 5 cases were present in patients with alarm features. This highlights the low yield of endoscopy in patients with dyspepsia and calls for a more conservative, nonendoscopic approach in management. This is especially true in patients without alarm features and younger than 55 years, where the prevalence of SEFs was 4.5%. While this could arguably be considered a significant percentage, none of these patients had malignancy. It is unlikely that treatment would significantly change in this small group of patients if they had undergone endoscopy, given that PPI and* H. pylori* testing are the mainstay of treatment. This study confirms the role of alarm features and age ≥ 55 in predicting the presence of SEFs. Patients with alarm features were more likely to have SEF compared to those with no alarm features (12.7 versus 5.4%, *p* = 0.004). Patients with both alarm features and age ≥55 had a 17% chance of having SEFs. Alarm feature and age ≥ 55 remained significant predictors of SEFs in multivariate analysis. In addition, we found that smoking is as useful in predicting SEFs as age ≥55 (aOR of 1.8 for both risk factors). Previous studies showed that smoking could increase the risk of peptic ulcerations [28–31]. Therefore, smoking could be considered an independent alarm feature and an important element in clinical decision making when stratifying patients with dyspepsia to undergo endoscopy. As expected, we found that combining several risk factors increased the chance of SEFs. For example, patients ≥ 55 years with any alarm feature had 4.2 (CI: 1.8–9.5) higher odds of having SEFs when compared to patients who were <55 years without alarm features (aOR 4.2).

In our study, we included patients with prior upper GI surgeries who are at risk of anastomotic complications such as marginal ulcers and anastomotic strictures [32–35]. Prior GI surgery was considered an alarm feature. Of 28 patients with prior upper GI surgery, 9 (32%) had significant findings (four anastomotic strictures, 3 anastomotic ulcers, one Fobi-ring erosion, and one candida esophagitis). This group of patients is at high risk of complications and endoscopy is always warranted to investigate upper GI symptoms.

Despite the low yield of endoscopy in outpatients with dyspepsia, a negative endoscopy can improve patient satisfaction and relieve anxiety due to fear of serious illnesses [36, 37]. In our study, the most common SEF was peptic ulcer disease (4%). We found lower prevalence of erosive esophagitis due to including patients with GERD only if they had accompanying dyspepsia symptoms. Our study found a low prevalence of malignancy in patients with dyspepsia (0.8%) which is comparable to previous studies (<0.5%) [13]. The study revealed a relatively lower prevalence of SEFs compared to other studies due to multiple factors. Previous studies were statistically heterogeneous [12, 14–19, 38]. Patients in our study were evaluated in the outpatient settings, given an appointment for their endoscopy, which usually takes several weeks to complete. In the meanwhile, they might be given a trial of PPI or asked to discontinue possible culprit medications, such as NSAIDs. This could have allowed the healing of some lesions and prevented their detection at the time of endoscopy. However, this is reflective of daily practice and should not be considered a weakness in this study.

Our study has several limitations. It had a retrospective design, was performed in a single center, and lacked cost analysis. In a randomized clinical trial, Laheij et al. randomized patients with dyspepsia referred to endoscopy to either prompt endoscopy followed by directed medical treatment versus empirical treatment with PPI followed by testing and treating* H. pylori* in the case of relapse [30]. They found that the empirical drug treatment resulted in less diagnostic endoscopies, lower costs, and equal effectiveness in the first year of follow-up. We did not collect data on celiac disease serologies in our patients because we do not routinely screen patients with dyspepsia for celiac disease in our practice. The prevalence of celiac disease in patients with dyspepsia varies between 0.5 and 2% [39]. This is expected to be much lower in our hospital where the majority of patients are African Americans (65%). Therefore, we do not think there were missed cases of celiac disease in our patient population.

Our study has several strengths. Despite our retrospective design, we were able to extract all information about patient demographics, symptoms and signs, and endoscopic findings from the medical record. Furthermore, we attempted to examine the utility of endoscopy in patients with dyspepsia in a more pragmatic setting rather than a randomized clinical trial. We did not find prior treatment with PPI or* H. pylori* status to be predictive of endoscopic findings, probably due to the low prevalence of peptic ulcer disease (4%). We also focused our study on outpatients with dyspepsia, as inpatients tend to have more severe symptoms and comorbidities, and therefore the approach to their management should be separate from outpatients. Finally, we chose to define “significant endoscopic findings” as one composite outcome that includes findings pertinent to the patients' management and related to their symptoms. We did not consider simple erosive or nonerosive gastroduodenal inflammation as a significant finding given that it is unlikely to contribute to the patients' symptoms or alter their long-term management. Previous studies showed poor association of these findings with dyspeptic symptoms [40, 41]. Erosive esophagitis class B and higher was considered significant, as it would require long-term maintenance treatment with PPI.

In summary, the prevalence of significant endoscopic findings in outpatients with dyspepsia is low, particularly in patients younger than 55 years and without alarm features. Guidelines should highlight the low yield of endoscopy in this group of patients and recommend nonendoscopic workup or empiric therapy as alternatives to endoscopy. Those patients should also be reassured that their symptoms are unlikely related to an underlying significant pathology and should be encouraged to defer endoscopy. The presence of any alarm feature, age ≥ 55, and smoking are all independent predictors of significant endoscopic findings. An approach to outpatients with dyspepsia that considers an age cutoff ≥ 55 and presence of any alarm features as indications for endoscopy is a simple and straightforward management strategy. However, a scoring system that considers multiple additional risk factors (such as smoking) is likely to improve the yield of endoscopy in dyspepsia and allow for a more accurate stratification of patients to endoscopic workup versus nonendoscopic workup or empiric therapy. Further studies are required to clarify the role of each alarm feature in predicting significant endoscopic findings and compile a more concise list of predictive risk factors to be used as a scoring system that guides clinical decision making.

## Figures and Tables

**Table 1:** Basics characteristics of outpatients with dyspepsia; Grady Memorial Hospital, Atlanta, Georgia, June 1, 2011–July 1, 2015.

Characteristic	*n* (650)	%
Age		
<55 years	433	66.6
≥55 years	217	33.4
Gender		
Female	473	72.8
Male	177	27.3
Race		
Black	423	65.1
Hispanic	112	17.2
White	57	8.8
Other	58	8.9
Smoking	161	24.8
Alcohol use		
None	456	70.2
Occasional	132	20.3
Heavy	62	9.5
Medications		
NSAIDs	504	77.5
PPI	456	70.2
H2-blocker	138	21.2
ASA	114	17.5
Other antiplatelets	7	1.1
Anticoagulant	5	0.8
*H. pylori* status prior to EGD		
Unknown	350	53.8
Positive and treated	140	21.5
Negative	126	19.4
Positive and not treated	34	5.2
Dyspepsia symptoms		
Epigastric pain	498	76.6
Nausea	280	43.1
Vomiting	170	26.2
Epigastric burning	138	21.2
Early satiety	79	12.2
Belching	34	5.2
Reflux symptoms		
Heartburns	172	26.5
Regurgitation	43	6.6
Alarm feature		
Vomiting	170	26.2
Weight loss	138	21.2
Anemia	103	15.8
Early satiety	79	12.2
Dysphagia	76	11.7
Previous peptic ulcer disease	41	6.3
Bleeding	38	5.8
Family history of GI cancer	29	4.5
Prior upper GI surgery	28	4.3
Previous GI cancer	12	1.8
Odynophagia	8	1.2
Lymphadenopathy or abdominal mass	4	0.6

NSAIDs: Nonsteroidal Anti-Inflammatory Drugs; PPI: proton pump inhibitor; ASA: aspirin; EGD: Esophagogastroduodenoscopy; GI: gastrointestinal.

**Table 2:** Endoscopic findings in outpatients with dyspepsia, stratified by alarm features; Grady Memorial Hospital, Atlanta, Georgia, June 1, 2011–July 1, 2015.

Characteristic	All patients		No alarm features		Any alarm feature		*p* value
	*n* (650)	%	*n* (223)	34.3%	*n* (427)	65.7%	
Any endoscopic abnormality	321	49.4	113	50.7	208	48.7	0.63
Significant endoscopic abnormality	66	10.2	12	5.4	54	12.6	0.004
Any peptic ulcer disease	26	4	3	1.3	23	5.4	0.01
Gastric	17	2.6	3	1.3	14	3.3	0.14
Duodenal	11	1.7	0	0	11	2.6	0.02
Gastritis							
Erosive	43	6.6	15	6.7	28	6.6	0.93
Nonerosive	193	29.7	74	33.2	119	27.9	0.16
Duodenitis							
Erosive	5	0.8	1	0.4	4	0.9	0.5
Nonerosive	47	7.2	14	6.3	33	7.7	0.54
Malignancy	5	0.8	0	0	5	1.2	0.1
Esophagitis							
Los Angeles class A	35	5.4	11	4.9	24	5.6	0.71
Los Angeles classes B, C, and D	16	2.5	5	2.2	11	2.6	0.79
Other significant endoscopic findings	25	3.8	5	2.2	20	4.7	0.12
Anastomotic stricture	4		0		4		
Candida esophagitis	4		0		4		
Anastomotic ulcer	3		0		3		
Severe hemorrhagic gastritis	3		2		1		
Barrett's esophagus	3		1		2		
Esophageal benign stricture	2		0		2		
Esophageal varices	2		1		1		
Extrinsic compression	1		0		1		
Gastric bezoar	1		0		1		
Paraesophageal hernia	1		1		0		
Fobi-ring erosion	1		0		1		
Other nonsignificant endoscopic findings	37	6	15	6.7	22	5.2	0.41
Benign polyps	31		12		19		
Nonobstructive Schatzki's ring	6		3		3		

LA: Los Angeles.

**Table 3:** Significant endoscopic findings in patients with and without alarm features stratified by age; Grady Memorial Hospital, Atlanta, Georgia, June 1, 2011–July 1, 2015.

Age	No alarm features	With any alarm feature	*p* value
<55	7/156 (4.5%)	28/277 (10.1%)	0.04
<40	1/64 (1.6%)	8/104 (7.7%)	NS
40–54	6/92 (6.5%)	20/173 (11.6%)	NS
≥55	5/67 (7.5%)	26/150 (17.3%)	0.045
Total	12/223 (5.4%)	54/427 (12.6%)	0.004

NS: not significant.

**Table 4:** Multivariate analysis of association of risk factors with significant endoscopic findings; Grady Memorial Hospital, Atlanta, Georgia, June 1, 2011–July 1, 2015.

Risk factor(s)	aOR (95% CI)	*p* value
Any alarm feature	2.3 (1.2–4.4)	0.01
Age ≥ 55	1.8 (1.1–3)	0.02
Smoking	1.8 (1.1–3.1)	0.03
Any alarm feature and age ≥ 55	4.2 (1.8–9.5)	0.0007
Any alarm feature and smoking	4.1 (1.8–9.4)	0.0005
Any alarm feature, age ≥ 55, and smoking	7.5 (2.9–19)	<0.0001

aOR: adjusted odds ratio. Final model included any alarm feature, age ≥ 55, and smoking. Race, gender, PPI use prior to endoscopy, *H. pylori* status, NSAIDs, and alcohol use had a nonsignificant association with endoscopic findings and were removed from the final model.

## Abbreviations

SEF: Significant endoscopic findings
GERD: Gastroesophageal reflux disease
*H. pylori*: * Helicobacter pylori*
PPI: Proton pump inhibitor
EGD: Esophagogastroduodenoscopy
NSAIDs: Nonsteroidal Anti-Inflammatory Drugs
ASA: Aspirin
GI: Gastrointestinal
LA: Los Angeles
GIST: Gastrointestinal stromal tumors
MALT: Mucosa associated lymphoid tissue
CI: Confidence interval
aOR: Adjusted odds ratio.
